# Intracerebroventricular Administration of Human Umbilical Cord Blood—Derived Mesenchymal Stem Cells Induces Transient Inflammation in a Transgenic Mouse Model and Patients with Alzheimer’s Disease

**DOI:** 10.3390/biomedicines10030563

**Published:** 2022-02-28

**Authors:** Su Hyeon Myeong, Hyeongseop Kim, Na Kyung Lee, Jung Won Hwang, Hee Jin Kim, Hyemin Jang, Soo Jin Choi, Duk L. Na

**Affiliations:** 1 Department of Health Sciences and Technology, SAIHST, Sungkyunkwan University, 81 Irwon-ro, Gangnam-gu, Seoul 06351, Korea; soarmsh@skku.edu (S.H.M.); jung89@skku.edu (J.W.H.); evekhj@gmail.com (H.J.K.); 2 Department of Neurology, Samsung Medical Center, Sungkyunkwan University School of Medicine, 81 Irwon-ro, Gangnam-gu, Seoul 06351, Korea; hmjang57@gmail.com; 3 Neuroscience Center, Samsung Medical Center, 81 Irwon-ro, Gangnam-gu, Seoul 06351, Korea; 4 Cell and Gene Therapy Institute (CGTI), Samsung Medical Center, 81 Irwon-ro, Gangnam-gu, Seoul 06351, Korea; hyeongseop09@gmail.com (H.K.); nakyunglee@skku.edu (N.K.L.); 5 Stem Cell Institute, ENCell Co., Ltd., Seoul 06072, Korea; 6 Alzheimer’s Disease Convergence Research Center, Samsung Medical Center, 81 Irwon-ro, Gangnam-gu, Seoul 06351, Korea; 7 School of Medicine, Sungkyunkwan University, Suwon 16419, Korea; 8 Biomedical Research Institute, R&D Center, MEDIPOST Co., Ltd., Seongnam 13494, Korea; sjchoi@medi-post.co.kr

**Keywords:** human-umbilical-cord-blood mesenchymal stem cells, intracerebroventricular administration, Alzheimer’s disease, inflammatory responses, fever

## Abstract

Previously we conducted a Phase I/IIa clinical trial in nine patients with mild to moderate Alzheimer’s disease (AD). Unexpectedly, all patients who were given injections of human-umbilical cord-blood-derived mesenchymal stem cells (hUCB-MSCs) developed fever which subsided after 24 h. Several possible causes of transient fever include bacterial infection, inflammatory reaction from the cell culture media composition, or the cells themselves. To delineate these causes, first we compared the levels of several cytokines in the cerebrospinal fluid (CSF) of AD patients who received saline (placebo) or hUCB-MSC injections, respectively. Compared to the placebo group, tumor necrosis factor-α (TNF-α), interleukin-1β (IL-1β), interleukin-6 (IL-6), and c-reactive protein (CRP) levels were increased in the hUCB-MSC group. Negative bacterial culture results of the CSF samples and the fact that the same hUCB-MSC administration procedure was used for both the placebo and hUCB-MSC groups ruled out the bacterial infection hypothesis. However, it was not yet clear as to whether the transplanted cells or the composition of the cell culture media generated the transient fever. Therefore, we carried out intracerebroventricular (ICV) injections of hUCB-MSCs in a 5xFAD mouse model of AD. Interestingly, we discovered that pro-inflammatory cytokine levels were higher in the hUCB-MSC group. Taken together, our data suggest that the cause of transient inflammatory response observed from both the clinical trial and mouse study was due to the transplanted hUCB-MSCs.

## 1. Introduction

Alzheimer’s disease (AD), the most common cause of dementia, is characterized by accumulation of amyloid beta, neurofibrillary tangle, and neuronal loss in the brain [1,2]. Following a long list of failed clinical trials [3], recently an amyloid vaccine (Aducanumab; Biogen Inc., Cambridge, MA, USA) has been approved by the U.S. Food and Drug Administration (FDA). The efficacy of the vaccine, however, remains controversial among researchers. Disease-modifying treatments that target a single mechanism or pathway may not be as effective as those that target multiple mechanisms due to the multifactorial pathogenesis of AD.

Mesenchymal stem cells (MSCs) have been appraised as a potent cell source for cell-based therapy. First, MSCs have been reported to be capable of homing to inflamed areas after transplantation [4]. Second, MSCs secrete paracrine factors that have therapeutic effects for AD [5]. MSCs secrete growth factors, chemokines, and cytokines to decrease amyloid beta [6], neurofibrillary tangle [7], neuroinflammation [8], and neuronal cell death [9]. In addition, they increase neurite outgrowth [10] and synaptic function [6,11]. Third, MSCs have immunomodulatory abilities. MSCs have been reported to improve Aβ pathology and memory impairment via activation of astrocytes and microglia [12].

The route to deliver MSCs into the brain is a critical issue that must be dealt with for AD MSC therapy. Previously, the intravenous [13], intra-arterial [14], intrathecal [15], intraparenchymal [16], and intracerebroventricular (ICV) [17,18] routes were explored as potential delivery routes. Out of the five routes, direct administration via the intraparenchymal or ICV routes seemed to be the most optimal [16,17,18]. Of the latter two routes, however, the ICV route may be advantageous over the intraparenchymal route because ICV administration (spinal taps included) is less invasive than parenchymal administration, especially when MSCs are injected repeatedly. Second, when MSCs are injected into the lateral ventricle, the cells adhere to the ventricular wall and then migrate towards the brain parenchyma [11,17]. Since the pathology of AD is widespread, ICV administration may be more effective since widespread delivery of the therapeutic agent is feasible.

Previously, we conducted a Phase I clinical trial, where nine AD patients received ICV injections of hUCB-MSCs via an Ommaya reservoir. Interestingly, all of the patients developed transient fever 6–12 h following ICV administration which lasted for about 24 h [19]. We hypothesized that the inflammatory reaction generated from either the chemical composition of the culture media or the MSCs themselves might have led to the fever. Therefore, the objectives of this current study were to, first, compare CSF cytokine levels from both the placebo and the hUCB-MSC groups, and second, to carry out in vivo experiments using an animal model to determine whether the inflammation originated from the culture media or the transplanted cells.

## 2. Materials and Methods

### 2.1. Preparation of hUCB-MSCs for Transplantation

The hUCB-MSCs (NEUROSTEM^®®^, MEDIPOST Co., Ltd., Seongnam, Gyeonggi-do, Korea) used in both the clinical trial and animal experiments were provided by MEDIPOST Inc (Biomedical Research Institute Co., Ltd., Seongnam, Gyeonggi-do, Korea) according to GMP guidelines. hUCB-MSC manufacture, cell quality control, and quality assurance were carried out in compliance with Korea Good Manufacturing Practices requirements by the MFDS. MSCs were isolated and characterized as reported previously [19,20,21,22,23]. Umbilical cord blood (UCB) was collected after receiving informed consent from all normal pregnant women. Mononuclear cells (MNCs) were first isolated from the cord blood using a Ficoll-Hypaque solution (Sigma-Aldrich Co., St. Louis, MO, USA). MNCs were collected, seeded in culture flasks, and cultured in 1× minimal essential medium alpha (α-MEM, Gibco, Carlsbad, CA, USA) containing 10% fetal bovine serum (FBS, Gibco, Waltham, MA, USA). Cells were passaged and the surface markers and differentiation potential of the cells [19,20,21,22,23] were assessed prior to use. hUCB-MSCs were cultured in 1× minimal essential medium alpha (MEM-α, Gibco- Thermo Fisher Scientific, Waltham, MA, USA) supplemented with 10% fetal bovine serum (FBS, Biowest, Riverside, MO, USA) and an antibiotic reagent gentamicin (Gibco- Thermo Fisher Scientific, Waltham, MA, USA). hUCB-MSCs (Passage 5) were cultured in a humidified 5% CO_2_ incubator at 37 °C. When hUCB-MSCs reached 80 to 90% confluency, cells were trypsinized, resuspended in phenol-red-free MEM-α and injected into the lateral ventricle.

### 2.2. CSF Collection from Alzheimer’s Disease Patients

The clinical study design was approved by the Korean FDA and the Institutional Review Board (IRB) of Samsung Medical Center, and the details of the clinical trial have been described previously [19]. In accordance with the guidelines approved by the IRB of the Samsung Medical Center, CSF samples were collected with informed consent from nine AD patients (Identifier: NCT02054208). We obtained CSF from nine mild to moderate Alzheimer’s disease dementia patients from Samsung Medical Center, Seoul, Korea, who had undergone the Phase I/IIa clinical trial. The patients underwent surgery for Ommaya reservoir implantation under local anesthesia. To analyze changes in inflammatory cytokine levels, CSF was collected from randomly chosen patients from the placebo (2 mL of normal saline per patient, total of three patients) and hUCB-MSC (3.0 × 10^7^ cells/2 mL per patient, total of six patients) groups (Table 1). Samples were collected before and 20 h after the first administration via an Ommaya reservoir.

### 2.3. Enzyme-Linked Immunosorbent Assay (ELISA) for CSF from Alzheimer’s Disease Patients

To measure levels of pro-inflammatory cytokine (TNF-α, IL-1β, IL-6, and CRP) expression in the CSF of AD patients, four different ELISA assays were performed (R&D Systems, Minneapolis, MN, USA). ELISA was carried out according to the manufacturer’s instructions. Optical density was measured at 450–570 nm using a xMark™ Microplate Absorbance Spectrophotometer (Bio-Rad Laboratories Inc., Hercules, CA, USA).

### 2.4. Experimental Animals

Six-month-old mice were used in this study. All animal experiments were reviewed and approved by the Institutional Animal Care and Use Committee (IACUC, Approval number: 20170223001, Date: 15 March 2017) of the Samsung Biomedical Research Institute (SBRI) at Samsung Medical Center. SBRI is an Association for Assessment and Accreditation of Laboratory Animal Care International (AAALAC International)-accredited facility and abides by the Institute of Laboratory Animal Resources (ILAR) guide. 5xFAD mice were purchased from Jackson Laboratory (Bar Harbor, ME, USA). The animals were housed under the following conditions: temperature (23 ± 3 °C), humidity (50 ± 20%), air ventilation 18–27 changes/h, and lighting cycle (12 h light–dark schedule).

### 2.5. Intracerebroventricular Cannulation and Injection of hUCB-MSCs in Mouse Models

We followed the standard brain cannulation procedure provided by the Jackson Laboratory (Bar Harbor, ME, USA). The cannula system consisted of a guide, dummy, internal cannula and screw (Plastics one technologies, Roanoke, VA, USA). Mice were placed on a rodent universal stereotactic frame (Harvard apparatus, Holliston, MA, USA) and anesthetized with 2% isoflurane (Hana Pharmaceutical Co., Ltd., Seoul, Korea) during the surgical procedure. The cannula was implanted into the right lateral ventricle (coordinates from bregma: A/P: −0.4 mm, M/L: +1.0 mm, and D/V: −2.3 mm) and mounted with dental cement to secure the position. One week after cannulation, hUCB-MSCs suspended in phenol-red-free MEM-α at a concentration of 2 × 10^5^/7 μL were injected via the cannula using a 25 μL Hamilton syringe (Hamilton Company, Reno, NV, USA). A total of 54 mice were used in this study: ELISA: *n* = 9 (sham), *n* = 9 (MEM-α) and *n* = 9 (hUCB-MSCs); histology: *n* = 9 (sham), *n* = 9 (MEM-α) and *n* = 9 (hUCB-MSCs).

### 2.6. Quantification of Pro-Inflammatory Cytokine Levels following hUCB-MSCs Administration in 5xFAD Mice

Frozen brain tissue samples were ground with a chilled mortar and pestle and then lysed with urea buffer (7 M urea, 2.8 M thiourea, 4% CHAPS, 130 mM dithiothreitol, and 40 mM Tris-HCl (pH 8.8)) and RIPA buffer (25 mM Tris-Cl (pH 7.6), 150 mM NaCl, 1% Sodium deoxycholate, 1% Triton X-100, 0.5% SDS) at a 1:1 ratio solution. After centrifugation at 13,000× *g*, 4 °C for 30 min, the supernatant was collected. The Bradford assay (Bio- Rad Laboratories Inc., Hercules, CA, USA) was performed to quantify protein amount and 100 ng of each sample was analyzed. ELISA assays for tumor necrosis factor-α (TNF-α; eBioscience, San Diego, CA, USA), interleukin-1β (IL-1β), interleukin-6 (IL-6) and c-reactive protein (CRP; R&D systems, Minneapolis, MN, USA) were conducted according to the manufacturer’s instruction.

### 2.7. Immunohistochemistry Analyses of the Mouse Brain Tissue

The harvested mouse brain tissues were fixed in 4% paraformaldehyde (PFA; Biosesang, Seongnam, Korea) for 24 h and then embedded in paraffin blocks. A total of 4 μm-thick coronal plane paraffin serial section slides were prepared using a microtome (Leica Biosystems, Wetzlar, Hesse, Germany). Slides were deparaffinized and citrate buffer solution (Dako, Glostrup, Denmark) was used for antigen retrieval. To check for successful transplantation of hUCB-MSCs, slides were stained using hematoxylin and eosin (H&E; BBC Biochemical, Mount Vernon, Washington, DC, USA). Slides were incubated in primary antibodies, anti-mouse STEM121 (1:500, Cellartis, Kusatsu, Shiga, Japan) and anti-rabbit cleaved caspase-3 (1:400, Cell Signaling Technology, Danvers, MA, USA) and then incubated in secondary antibodies (Cy3 conjugated goat anti-mouse and 488 conjugated goat anti-rabbit, Jackson ImmunoResearch, Europe Ltd., Newmarket, UK) for 1 h at room temperature. After PBS washing, slides were mounted using Gel/mount™ (Biomeda Corp., Foster City, CL, USA). Fluorescent microscopic images were acquired using a confocal microscope (Carl Zeiss AG, Jena, Germany).

### 2.8. Data Analysis

Data analysis was performed using Prism v5.0 (GraphPad Software, San Diego, CA, USA). All values are represented as average ± S.E.M of independent experiments. The *t*-test was used to compare the results of four inflammatory cytokine levels before and after administration in both the placebo and hUCB-MSC groups. One-way ANOVA was used to compare the inflammatory cytokine levels of the MEM-α and hUCB-MSC groups (3, 9, and 24 h) to the sham group. *p*-values of <0.05 were considered statistically significant for all analyses.

## 3. Results

### 3.1. The Level of Pro-Inflammatory Proteins Was Increased after hUCB-MSCs Were Administered into the Lateral Ventricle of AD Patients

The baseline characteristics of the placebo group (*n* = 3) and hUCB-MSC group (*n* = 6) are shown in Table 1. There were no differences in age (placebo 61.3 years vs. hUCB-MSCs 64.5 years), gender—male (M): female (F) (placebo 2:1 vs. hUCB-MSCs 3:3), education years (placebo 16.3 years vs. hUCB-MSCs 10.2 years), MMSE score (placebo 23 vs. hUCB-MSCs 21) and APOE 4 carrier frequency (placebo 50% vs. hUCB-MSCs 42%) between the two groups. All patients were positive for amyloid PET. Signs of fever were not observed from participants who received saline administrations (placebo group). In contrast, AD patients who received administrations of hUCB-MSCs showed signs of fever starting at 3 h post administration, reaching its peak level by 6 to 12 h. The fever subsided 36 h post antipyretics administration. Details of the clinical findings including the fever pattern have been described elsewhere [19]. Due to the occurrence of fever, levels of fever-related pro-inflammatory cytokines were assessed from the CSF samples.

**Figure 1:**
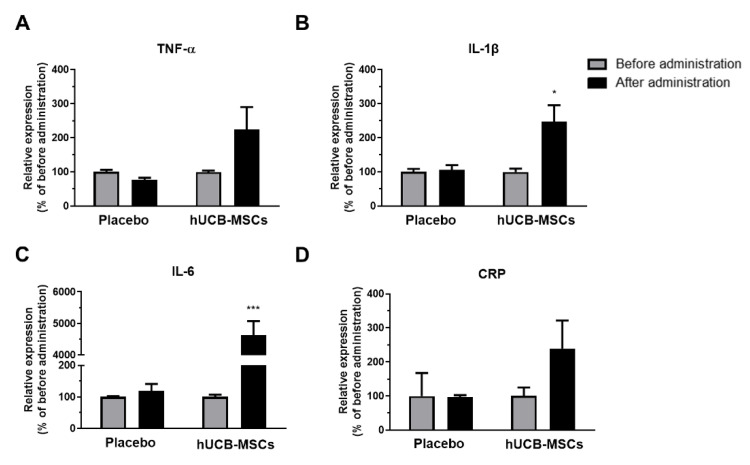
Pro-inflammatory cytokine levels increased in AD patients following hUCB-MSCs injection. Tumor necrosis factor-α (TNF-α), interleukin-1β (IL-1β), interleukin-6 (IL-6), and c-reactive protein (CRP) ELISA was carried out using CSF samples collected before and after administration of saline (placebo, *n* = 3) or hUCB-MSCs (*n* = 6). (**A**) TNF-α, (**B**) IL-1β, (**C**) IL-6, and (**D**) CRP expression levels increased after hUCB-MSCs administration. In the placebo group, there was no difference in expression of all four cytokines before and after administration (* *p* < 0.05, *** *p* < 0.0001). The data are presented as the mean ± S.E.M.

Four representative cytokine markers were measured by ELISA using CSF samples collected before and after administration of saline or hUCB-MSCs. Following hUCB-MSC administration, tumor necrosis factor-α (TNF-α) levels increased by about 2.3-fold (Figure 1A), while interleukin-1β (IL-1β) and interleukin-6 (IL-6) levels increased significantly by about 2.6-fold (Figure 1B) and 43.2-fold (Figure 1C), respectively. C-reactive protein (CRP) levels increased by about 3.5-fold (Figure 1D).

### 3.2. Pro-Inflammatory Protein Levels Were Increased after Administration of hUCB-MSCs into the Lateral Ventricle of the Mouse Model of AD

**Figure 2:**
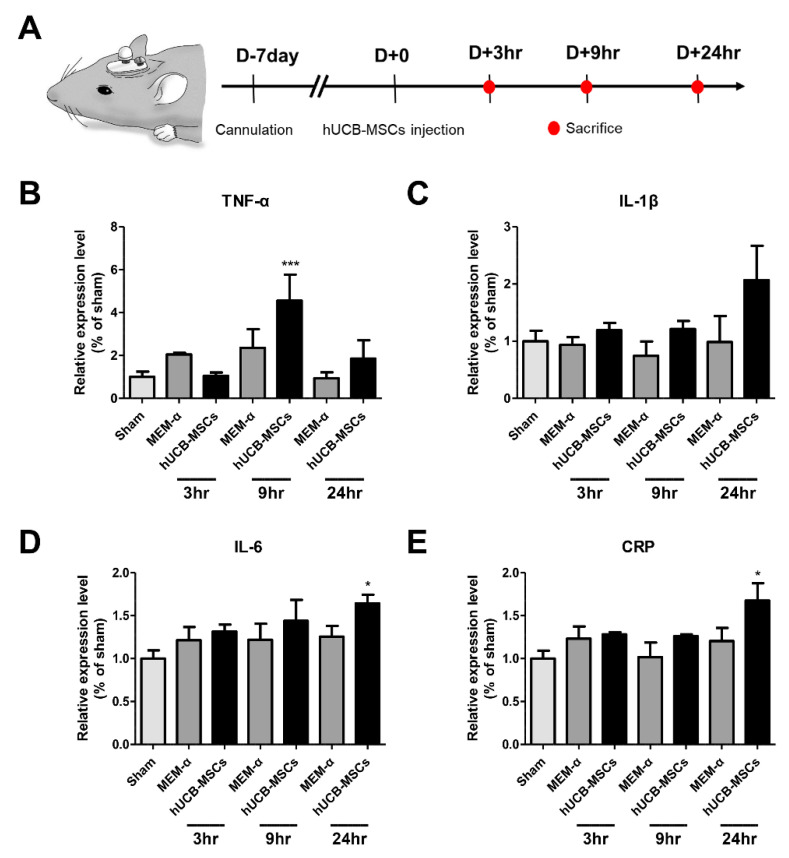
Pro-inflammatory cytokine levels increased in 5xFAD mice following hUCB-MSCs administration. (**A**) Mouse experiment timeline. (**B**) TNF-α, (**C**) IL-1β, (**D**) IL-6, and (**E**) CRP expression levels were analyzed at three different time points: 3, 9, and 24h after hUCB-MSC administration. TNF-α expression levels increased 9 h after hUCB-MSCs administration and IL-1β, IL-6 and CRP expression levels increased 24 h after hUCB-MSC administration (* *p* < 0.05, *** *p* < 0.00001). The data are presented as mean ± S.E.M (average of three independent experiments).

To investigate the cause of the inflammation observed in AD patients transplanted with hUCB-MSC, we conducted an additional experiment using the 5xFAD mouse model. We assessed whether the inflammation was due to the culture media or the cells themselves. In order to reduce the inflammatory response that may arise from surgical procedure, hUCB-MSCs were administered a week after the cannulation (Figure 2A). The sacrifice time points were 3, 9, and 24 h, which were equivalent to the time points where fever was observed from the AD patients. TNF-α levels increased significantly by about 4.6-fold in the 9 h hUCB-MSC group compared to the sham group (Figure 2B). IL-1β levels increased by about 2.1-fold in the 24 h hUCB-MSC group compared to the sham group (Figure 2C). IL-6 and CRP levels were significantly increased by about 1.6- and 1.7-fold in the 24 h group, respectively, when compared to that of the sham group (Figure 2D,E).

### 3.3. hUCB-MSCs Adhered to the Lateral Ventricle Wall after ICV Administration in the Mouse Brain

**Figure 3:**
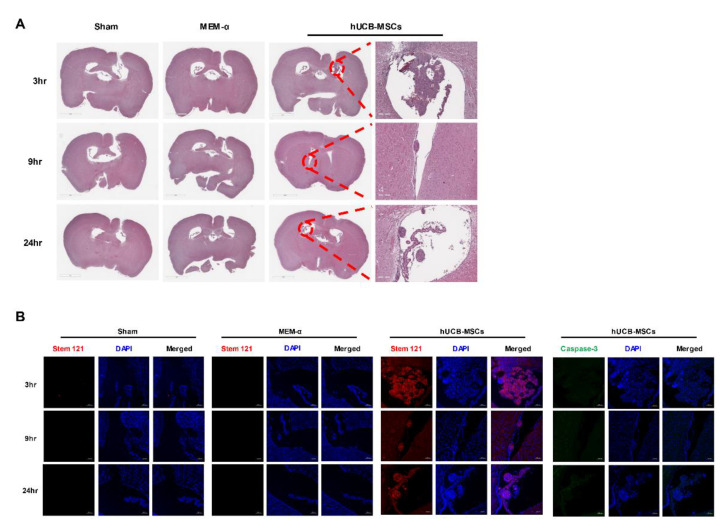
Adherence of hUCB-MSCs to the ependyma cell wall of the mouse brain. (**A**) Transplantation of hUCB-MSCs into the lateral ventricle of 5xFAD mouse is shown via H&E staining. hUCB-MSCs were observed only from the hUCB-MSC groups sacrificed at the 3, 9, and 24 h post transplantation. Adherence of hUCB-MSC aggregates to the ependymal cell layer was observed. (**B**) The cell aggregate was identified as the transplanted human MSCs using the human-cell-specific marker, STEM 121 (red signal) and cell death marker, caspase-3 (cleaved caspase-3, green signal). H&E: Scale bar = 2 mm (**left**), 200 μm (**right**), IHC: scale bar = 100 μm.

After hUCB-MSCs were administered into the lateral ventricle of 5xFAD mice, the presence and distribution of transplanted hUCB-MSCs were examined using H&E staining (Figure 3A). Attachment of hUCB-MSCs to the ependymal cell layer was observed in the hUCB-MSC groups sacrificed at both 9 and 24 h. The cell aggregate was further analyzed using STEM 121, a marker known for detecting human cells (Figure 3B). In addition, to confirm the viability of the remaining cells positive in Stem 121, it was analyzed using a cleaved caspase-3 marker. As a result, no cell death was observed in hUCB-MSCs within 24 h of administration to the ventricle (Figure 3B).

## 4. Discussion

In our previous Phase I/IIa clinical trial, AD patients unexpectedly experienced transient fever following hUCB-MSC administration which subsided after 24 h [19]. Previous studies have also reported on cases where several patients developed transient fever following administration of human MSCs [24,25]. Significant differences in expression levels of pro-inflammatory cytokines (TNF-α, IL-1β, IL-6 and CRP) were not observed when assessing CSF samples collected before and after saline administration for the placebo group. In contrast, pro-inflammatory cytokine levels increased by more than two-fold following hUCB-MSCs administration in AD patients (Figure 1). Out of the four cytokines, levels of IL-6 were significantly increased by about 43-fold following administration of hUCB-MSCs. IL-6 is known as a pyrogenic cytokine and plays an important role in fever. IL-6 also induces migration of lymphocytes [26]. Thus, the remarkable increase in circulating IL-6 levels may have played an influential role in generating the fever in the patients. However, since hUCB-MSCs were suspended in MEM-α, we cannot exclude the possibility that the cause of fever was due to the media itself. Hence, we carried out an additional experiment to determine whether the cause of fever was MEM-α or hUCB-MSCs.

Compared to the sham (only the cannula was inserted) and MEM-α groups, pro-inflammatory cytokine levels were only increased from the hUCB-MSC group. This strongly suggested that the hUCB-MSCs and not the MEM-α culture media may have contributed towards generating the inflammatory response (Figure 2). These results are consistent with the results from our previous animal studies. For example, when syngeneic, allogeneic, and xenogeneic adipose MSCs were injected into the parenchyma of wild-type mice, high levels of leukocyte infiltration were detected in the xenogeneic group followed by the allogeneic group [16]. In another beagle study, stromal edema was observed following inflammatory cell infiltration on both sides of the choroid plexus a week after administration of hUCB-MSCs via the ICV route. In addition, neutrophil levels in the beagle CSF increased at 24 h following administration and decreased sequentially after 4 and 7 days [17]. In the clinical trial, WBC count in the CSF was normalized 4 weeks after administration of hUCB-MSCs [19].

To determine changes in pro-inflammatory cytokine levels at the same time, fever was observed from the AD patients (3 h, 9 h, and 24 h when the fever subsided), an animal experiment was conducted. More than a 1.5-fold increase in pro-inflammatory cytokine levels (TNF-α, IL-1β, IL-6 and CRP) were observed from the brain tissue lysate of 5xFAD mice that received hUCB-MSCs administration (Figure 2). Endogenous pyrogens such as TNF-α, IL-1β, and IL-6 are known as pro-inflammatory cytokines that possess a synergistic relationship. TNF-α induces fever and IL-1 and IL-6 production, which generates an inflammatory response [27]. CRP is also known to be mainly induced by IL-6 expression rather than TNF-α and IL-1 [28]. Based on this notion, TNF-α was the first to be expressed among the four cytokines at 9 h post hUCB-MSCs administration, consequentially inducing IL-1, IL-6, and CRP production. According to the IHC results, adherence of MSCs to the ependyma wall was observed from the hUCB-MSC administered groups sacrificed at 9 and 24 h (Figure 3). The transient fever may have been brought about by the increase in inflammatory responses possibly initiated via the interaction between the MSC aggregates and the ependymal cell wall. Further study, however, is warranted.

This study has several limitations. First, due to differences in the species of the recipient, hUCB-MSC transplantation was allogeneic in clinical trials while xenogeneic in the animal experiments of this study. Thus, our animal experiments did not perfectly simulate the clinical setting. Second, for the analysis of the pro-inflammatory cytokines, we used CSF samples from the AD patients, whereas brain lysates were analyzed in the animal experiment. Due to the small size of the mouse brain, it was difficult to collect the CSF samples for analysis. Third, the mouse equivalent dose of the cell concentration used for the clinical trial was not applied to this study. As a preliminary study, a total of 1.0 × 10^5^ cells suspended in 7 μL of media (mouse equivalent dose of the human dose) was administered into the lateral ventricle of mice. Interestingly, signs of inflammatory responses were not noted (data not shown). Therefore, a higher concentration of hUCB-MSCs was transplanted into the lateral ventricle of mice for this study to closely mimic the inflammatory responses that have arisen from the clinical trials. The fourth limitation of this study was that the chemotactic roles and potential positive effects that may arise from inflammation were not extensively looked into in this study. When transient inflammation occurs, growth factors such as cytokines are released. Although a transient increase in the expressions of fever-related inflammatory cytokines such as TNF-α and IL-1β were observed following MSC administration, these cytokines may also increase the chemotactic properties of MSCs [29,30]. The last limitation of this study was that an extended follow-up was not conducted. Observations were made up to 24 h in this study because fever subsided after 24 h in the clinical trial. Furthermore, according to studies that we have published recently, very few residual human MSCs were identified in the mouse brain one week following MSC transplantation [16,31,32]. Thus, although not extensively studied, since very few residual human MSCs were present in the mouse brain, signs of inflammatory responses may not have been detected at 1 or even 2 weeks following MSC transplantation.

While PET imaging has been used to determine amyloid positivity in our clinical trial, it can also be utilized to identify inflamed regions in the brain. For future studies, it would be useful to utilize glucose PET or PET scans imaged with inflammation biomarkers such as the translocator protein (TSPO) ligand, COX-2, or TNF-α [33,34] when they are commercialized, to possibly clarify the cause of inflammatory responses generated following MSC transplantation.

## 5. Conclusions

The results of this study strongly suggest that hUCB-MSCs, and not the culture media, elevated cytokine levels, which may have given rise to the fever observed from AD patients who received hUCB-MSC administrations. Further study is required to determine methods to reduce fever and inflammatory responses following MSC transplantation in order to enhance the therapeutic efficacy of MSC AD therapy.

## Figures and Tables

**Table 1:** Baseline characteristics of AD patients.

Subject	Age	Gender	Education Years	MMSE Score	APOE Genotype	Florbetaben PET
Placebo 1	60	M	21	21	ε3/ε3	+
Placebo 2	59	F	12	22	ε3/ε4	+
Placebo 3	65	M	16	26	ε4/ε4	+
hUCB-MSCs 1	66	F	6	20	ε3/ε4	+
hUCB-MSCs 2	53	M	16	21	ε3/ε3	+
hUCB-MSCs 3	73	M	12	23	ε4/ε4	+
hUCB-MSCs 4	65	M	12	20	ε3/ε4	+
hUCB-MSCs 5	65	F	6	19	ε3/ε4	+
hUCB-MSCs 6	65	F	9	23	ε3/ε3	+

## Data Availability

The data presented in this study are available on request from the corresponding author.
